# Half-leaf width symmetric distribution reveals buffering strategy of *Cunninghamia lanceolata*

**DOI:** 10.1186/s12870-021-03000-x

**Published:** 2021-05-17

**Authors:** Xi Peng, Meifang Zhao, Shuguang Liu, Wende Yan

**Affiliations:** 1 College of Life Science and Technology, Central South University of Forestry and Technology, Changsha, 410004 Hunan China; 2 Huitong National Field Station for Scientific Observation and Research of Chinese Fir Plantation Ecosystem in Hunan Province, Huitong, 410015 China; 3 National Engineering Laboratory for Applied Forest Ecological Technology in Southern China, Changsha, 410004 China

**Keywords:** Leaf buffering strategy, Leaf width, Leaf length, Taper model, Regulation mechanism, Leaf biomechanics, Leaf morphology, Conifer

## Abstract

**Background:**

Leaf length and width could be a functioning relationship naturally as plant designs. Single-vein leaves have the simplest symmetrical distribution and structural design, which means that fast-growing single-vein species could interpret the scheme more efficiently. The distribution of leaf length and width can be modulated for better adaptation, providing an informative perspective on the various operational strategies in an emergency, while this mechanism is less clear. Here we selected six age groups of *Cunninghamia lanceolata* pure forests, including saplings, juveniles, mature, and old-growth trees. We pioneered a tapering model to describe half-leaf symmetric distribution with mathematical approximation based on every measured leaf along developmental sequence, and evaluated the ratio of leaf basal part length to total length (called tipping leaf length ratio).

**Results:**

The tipping leaf length ratio varied among different tree ages. That means the changes of tipping leaf length ratio and leaf shape are a significant but less-noticed reflection of trees tradeoff strategies at different growth stages. For instance, there exhibited relatively low ratio during sapling and juvenile, then increased with increasing age, showing the highest value in their maturity, and finally decreased on mature to old-growth transition. The tipping leaf length ratio serves as a cost-benefit ratio, thus the subtle changes in the leaf symmetrical distribution within individuals reveal buffering strategy, indicating the selection for efficient design of growth and hydraulic in their developmental sequences.

**Conclusions:**

Our model provides a physical explanation of varied signatures for tree operations in hydraulic buffering through growth stages, and the buffering strategy revealed from leaf distribution morphologically provides evidence on the regulation mechanism of leaf biomechanics, hydraulics and physiologies. Our insight contributes greatly to plant trait modeling, policy and management, and will be of interest to some scientists and policy makers who are involved in climate change, ecology and environment protection, as well as forest ecology and management.

**Supplementary Information:**

The online version contains supplementary material available at 10.1186/s12870-021-03000-x.

## Background

Plants constantly buffer biophysical stress, mechanical resistance, and other environmental impacts for better adaptation and evolution throughout life stages [1, 2]. The vascular tapering patterns revealed the buffering strategy is helpful to understand the hydrodynamical, biomechanical and geometrical design of plants based on the application of resource distribution theory under global warming trends [3]. This is mostly confirmed in roots, trunks, and branches of broad-leaved species, such as bamboo [4–7]. However, leaves acting as a transfer with external water vapor exchange constitute a substantial (up to 60%) part of the resistance to water flow through plants, and thus influence transpiration, photosynthesis, and productivity [8]. Thus, the leaves seem to be more relevant for a sophisticated and effective hydraulic buffering strategy, exactly as leaf shape is finely tuned to adapt photosynthetic efficiency and hydraulic architecture [9]. The exploration of this phenomenon should therefore enhance our understanding of leaf design, plant architecture, and the hydraulic system of a self-similar fractal response to future climate change [2, 10–13].

Plant vascular tapering is demonstrated as conduits narrow in diameter along branch or root segments, while, how leaf width varies in leaf length segments is less concerned. Most studies have focused on which leaf vein tapering (e.g., midrib or veinlet) allows to decrease the path resistance and increase hydraulic capacity relative to construction costs [14–16]. But the leaf width shows tremendous variation based on similar vein tapering mechanisms [7, 17, 18]. Significantly, leaf width contributes more constraints to hydraulic design, because the maximum mesophyll hydraulic pathway is determined by leaf width [14, 19]. Thus, it is universally accepted that broadleaves hold more complicated transport network or higher vein density to meet the wider structural construction, experiencing substantial evolutionary advantages, while the needles are cylindrical and take only one vein [19–21]. For single-vein leaves, mechanical reinforcement and moderate physiological process are on the strength of no more than one porous transport pipe without ‘plan B’ as a consequence [22]. Nevertheless, higher costs in leaf width might incur higher costs in water-sourcing root biomass to supply the transpiration [23], either a direct reduction in net carbon gain and competitive growth, leading to a competitive disadvantage [24]. Like some needle species put in a pioneer and high productivity role than broad-leaved species in the same climate regime [25], there should be a kind of leaf with only one vein to support moderate width, and further, still sustain high productivity. Additionally, traditional coarse data of simply leaf maximum or estimated mean width are not as accurate for predicting leaf hydraulic buffering, encouraging us to evaluate width along with length segments and simulate leaf distribution trajectory more precisely.

Taxonomists proposed that there was a simplest way to differentiate orbiculate leaf species, was to locate the axis or, in some cases, the zone of greatest leaf width that lay perpendicular to the axis of the greatest length (long axis) [26]. The leaf is divided into two parts (basal part and distal part) depending on the zone [27]. Such as the tipping leaf length ratio (the ratio of basal part length to greatest length) is 2/5 in ovate leaves, and 1/3 in oblong leaves theoretically [26]. Leaf broadens to contain more structural buildings with an expansion of leaf length, and meets one part to stop widening, with leaf margin converging therewith. The tipping leaf length ratio represents that leaf reaches to the highest axial transport capacity and radial resistance. For quick adaptation, each leaf inherits the basal part length ratio, and modifies it within a shoot or crown, resulting in intraspecific differences [28]. However, less concentration has been devoted to exploring the temporal and spatial variation of such ratio. Whatever broadleaves or narrower leaves, different tipping leaf length ratio of leaves might be contributed to different functions of biomechanics and bioengineering, serving as the signal of buffering strategy, indicating developmental plastically superiorities [29–33].

Early predictors were devoted to testing the appropriate functionality and optimal cutting age of high yield and rapid growth for the selection of commercial trees in a breeding program. Fast-growing trees tend to be associated with elevated transpiration rates and higher hydraulic efficiency [34], thus sensible hydrodynamic variation performances were easier discovered comparing to slow-growing species. For instance, *Cunninghamia lanceolata* as a representative species, is the most prevalent and widely distributed commercial evergreen in China. It has a long history of cultivating species for fast-growing and high productivity [35, 36]. Besides, many forest management experience can provide us with the differentiation of growth stages and growth states.

**Fig. 1:**
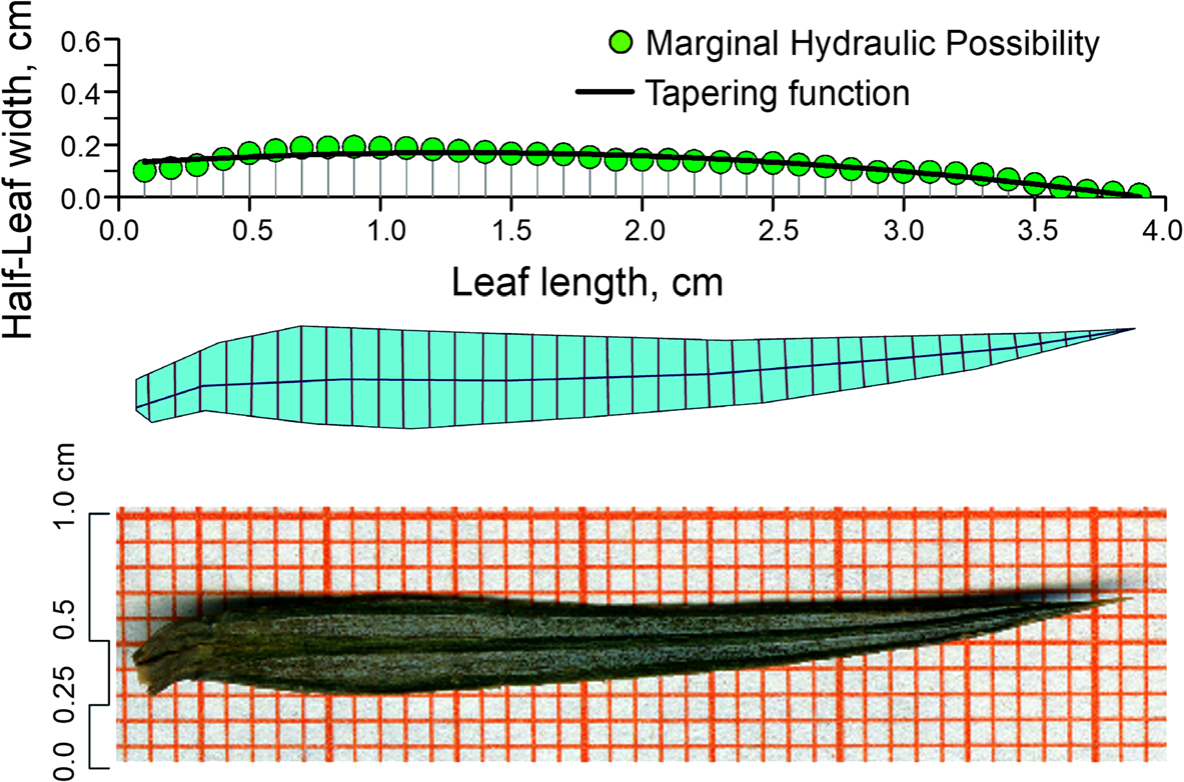
A general model of leaf length and half-leaf width quadratic relationship, taken one *C. lanceolata* leaf as an example here (more details are shown in supporting information Fig. S1). The leaf width of needles universally ranges from 0.04 to 0.15 cm, while broad leaves perform much wider (greater than 1 cm) [37, 38]. Further, the modeled species *C. lanceolata* show somewhere in between. The images on the top, middle, below are the half-leaf width model, the contour extraction image and the original scanned image, separately. The data of the model come from the middle image, and blue line means leaf length between leaf petiole to tip along leaf midrib, red lines mean leaf widths at every 0.1 cm length segments. Green dots in the model represent half-leaf width marginal hydraulic possibility, and dark linear shows the leaf tapering function between half-leaf width(Y) and leaf length(X), which could be fitted as: ***Y = C + B***_**1**_***X + B***_**2**_***X***^**2**^. Diameters are shown in below image

Distribution of orbiculate leaf length and width diversity could be quantified with relevant characters and mathematical approximations (Fig. 1). According to most leaf length and half-leaf width variations, they might be functional relationships naturally as plant designs. There is possible to be many parabolic curves differing in skewness and kurtosis. It is intermediate between broad leaves and needles with moderate leaf width, as well as the conclusion drawn from their simple symmetric distribution can be generalized and followed. The symmetric distribution demonstrates the tipping length ratio is the largest leaf length divided by horizontal distance where derivative of function is zero. Here, we propose that: (1) the tipping leaf length ratio might be delicately varied in leaves within species; and (2) buffering strategy would be caught in leaves according to the half-leaf distribution variation in different developmental stages. Our specific objectives are to: (1) construct a tapering model of leaf length and half-leaf width, predicting tipping leaf length ratio from leaf trajectory function, and take *C. lanceolata* as an example; (2) evaluate the models of half-leaf symmetric model in terms of its predictive power and applicability to unexplored leaf designations of buffering strategy and growing stages of development; and (3) discuss the possible constraints of leaf biomechanics and physiologies in hydraulic buffering.

## Results

**Fig. 2:**
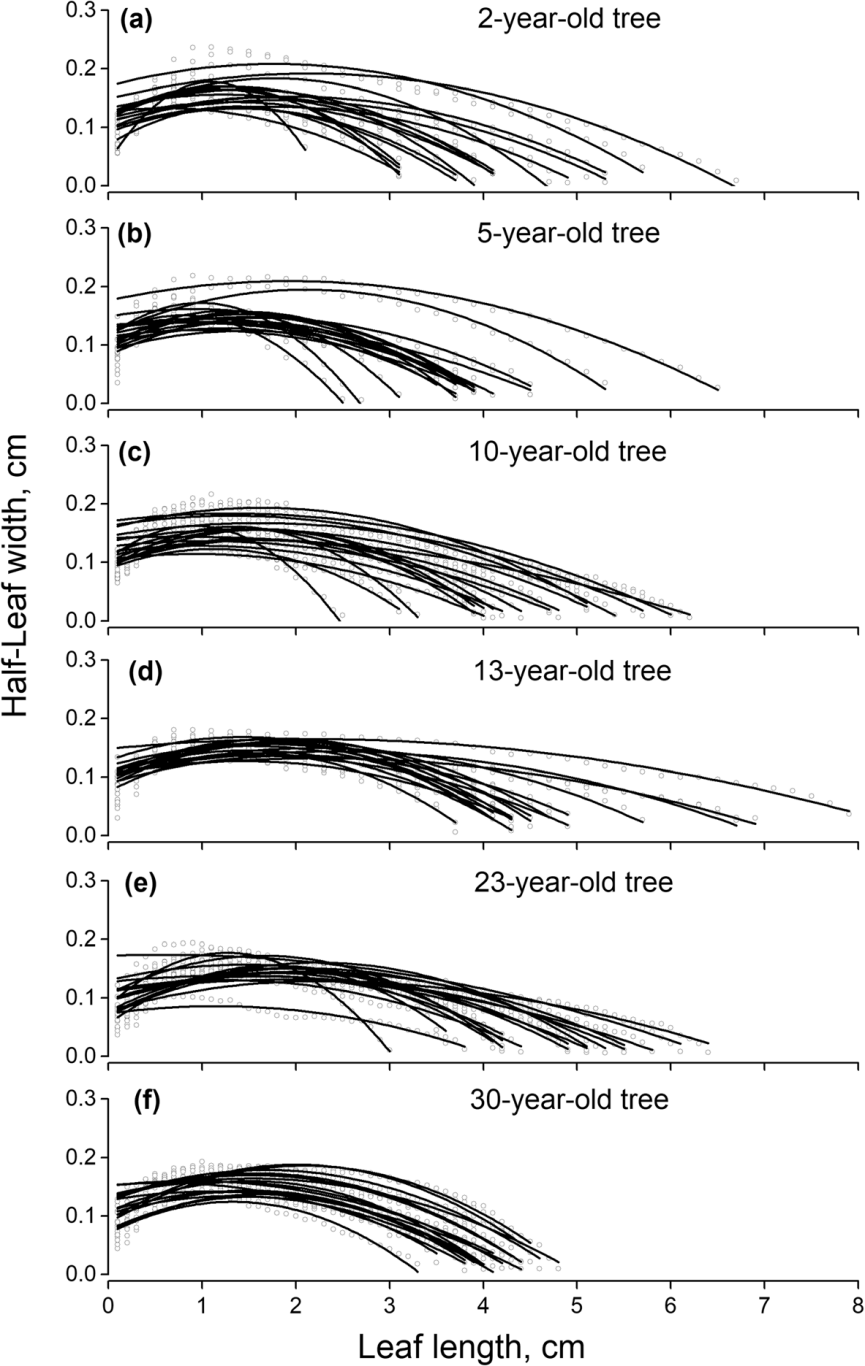
There are 108 models based on 3596 groups of data between different tree age stages of *C. lanceolata*. Models are fitted as: ***Y = C + B***_**1**_***X + B***_**2**_***X***^**2**^ (regression parameters of relationships are shown in supporting information Table S1)

Means of leaf width varies from 0.222 ± 0.08 cm for 23-yr-old, to 0.242 ± 0.084 cm for 30-yr-old among tree age gradients, and averages 0.234 cm in total (Fig. 2, Table 1). The 23-yr-old trees (0.222 ± 0.08 cm) show significant lowest leaf width among the studied six age group trees while the 30-yr-old (0.242 ± 0.084 cm) shows significant highest value comparing to the rest five age groups (*p* < 0.05). The trees in age from 2- to 13-yr-old show medium leaf width ranging from 0.252 cm to 0.247 cm (more details are shown in Table 1).

**Table 1:** Statistical description of leaf width, maximum leaf width, leaf length and half-leaf tipping length ratio in tree age groups, using mean, standard deviation, standard error, 95% confidential intervals (lower and upper value), median, minimum and maximum value

Leaf parameter	Tree age (year)	N*	Mean ± Standard Deviation**	Standard Error	CV%***	95% Confidence Interval	
						[Lower, Upper]	Median [Minimum, Maximum]
Width (cm)	2	386	0.241 ± 0.098 ab	0.005	40.664	[0.231, 0.251]	0.252 [0.002, 0.473]
	5	367	0.234 ± 0.093 ab	0.005	39.744	[0.225, 0.244]	0.24 [0.004, 0.437]
	10	804	0.234 ± 0.09 a	0.003	38.462	[0.228, 0.24]	0.246 [0.01, 0.434]
	13	452	0.23 ± 0.081 ac	0.004	35.217	[0.223, 0.238]	0.247 [0.005, 0.361]
	23	861	0.222 ± 0.08 c	0.003	36.036	[0.217, 0.228]	0.236 [0.012, 0.388]
	30	740	0.242 ± 0.084 b	0.003	34.711	[0.236, 0.249]	0.258 [0.013, 0.387]
	Total	3610	0.233 ± 0.087	0.001	37.339	[0.23, 0.236]	0.246 [0.002, 0.473]
Maximum leaf width (cm)	2	18	0.346 ± 0.055 a	0.013	16.021	[0.319, 0.374]	0.335 [0.277, 0.473]
	5	18	0.330 ± 0.042 ab	0.010	12.699	[0.310, 0.351]	0.326 [0.277, 0.437]
	10	18	0.331 ± 0.048 ab	0.011	14.628	[0.307, 0.356]	0.326 [0.262, 0.434]
	13	18	0.320 ± 0.026 ab	0.006	8.096	[0.307, 0.333]	0.328 [0.266, 0.361]
	23	18	0.334 ± 0.034 ab	0.008	10.176	[0.317, 0.351]	0.334 [0.278, 0.387]
	30	18	0.310 ± 0.040 b	0.009	12.925	[0.290, 0.330]	0.311 [0.204, 0.388]
	Total	108	0.329 ± 0.043	0.004	12.970	[0.321, 0.337]	0.327 [0.204, 0.473]
Length (cm)	2	18	4.32 ± 1.075 ab	0.253	24.884	[3.785, 4.854]	4.240 [2.881, 6.823]
	5	18	3.977 ± 0.904 a	0.213	22.731	[3.527, 4.426]	3.95 [2.433, 6.421]
	10	18	4.518 ± 0.99 ab	0.233	21.912	[4.025, 5.01]	4.311 [2.76, 6.158]
	13	18	4.887 ± 1.169 b	0.276	23.921	[4.306, 5.469]	4.430 [3.702, 7.826]
	23	18	4.484 ± 0.899 ab	0.212	20.049	[4.036, 4.931]	4.690 [2.879, 6.092]
	30	18	4.024 ± 0.424 a	0.100	10.537	[3.814, 4.235]	4.110 [3.225, 4.887]
	Total	108	4.368 ± 0.97	0.093	22.207	[4.183, 4.553]	4.243 [2.433, 7.826]
Half-leaf tipping length ratio	2	18	0.34 ± 0.051 ab	0.012	15.000	[0.315, 0.366]	0.348 [0.191, 0.425]
	5	18	0.328 ± 0.075 ab	0.018	22.866	[0.291, 0.365]	0.345 [0.183, 0.41]
	10	18	0.303 ± 0.08 a	0.019	26.403	[0.264, 0.343]	0.319 [0.123, 0.409]
	13	18	0.365 ± 0.051 bc	0.012	13.973	[0.34, 0.391]	0.362 [0.272, 0.441]
	23	18	0.381 ± 0.104 c	0.024	27.297	[0.33, 0.433]	0.408 [0.1, 0.525]
	30	18	0.361 ± 0.106 c	0.025	29.363	[0.308, 0.414]	0.358 [0.155, 0.593]
	Total	108	0.347 ± 0.083	0.008	23.919	[0.331, 0.362]	0.352 [0.1, 0.593]

* N = sample size. **The differences of the means between tree age groups were tested using a One-Way ANOVA test (Fisher LSD test); different letters denote statistically significant (*p* < 0.05) differences between the means. *** CV = coefficient of variation

*C.lanceolata* half-leaf symmetric distributions show one widest part along leaf axis, and successfully demonstrate general quadratic growth functions (Fig. 2, Table S1). The variations of half-leaf tapering trajectories of *C.lanceolata* among different tree age groups are shown in Fig. 2. No logarithmic scale is used for better demonstrations of leaf length and half-leaf width trends. The leaves show a similar general pattern, with leaf length explaining 71.7 to 98.7% (averaged in 89.9%) of the total half-leaf width variation. And the adjustment coefficient of determination (Adj. R^2^) of 58.3% of all models is above 90% (Table S1). Half-leaf width rises to the apex (the widest part of half-leaf width averages 0.165 cm) then converges to maximum leaf length (averages 4.368 cm) (More details are shown in Table 1). The final model of leaf length (*x*) and half-leaf width (*Y*) of each age group trees is as following:

2-yr-old tree: *Y* = (0.142 ± 0.005) + (0.005 ± 0.004)*x* + (−0.005 ± 0.0008)*x*^2^ (R^2^ = 0.357, *P* < 0.001)

5-yr-old tree: *Y* = (0.147 ± 0.005) + (−0.008 ± 0.005)*x* + (−0.002 ± 0.0009)*x*^2^ (R^2^ = 0.275, *P* < 0.001)

10-yr-old tree: *Y* = (0.142 ± 0.003) + (−0.006 ± 0.003)*x* + (−0.005 ± 0.0005)*x*^2^ (R^2^ = 0.498, *P* < 0.001)

13-yr-old tree: *Y* = (0.138 ± 0.004) + (−0.001 ± 0.003)*x* + (−0.002 ± 0.0004)*x*^2^ (R^2^ = 0.376, *P* < 0.001)

23-yr-old tree: *Y* = (0.119 ± 0.003) + (0.020 ± 0.002)*x* + (−0.007 ± 0.0004)*x*^2^ (R^2^ = 0.542, *P* < 0.001)

30-yr-old tree: *Y* = (0.120 ± 0.003) + (0.040 ± 0.003)*x* + (−0.014 ± 0.0007)*x*^2^ (R^2^ = 0.583, *P* < 0.001)

Leaf length shows a definite variation except in 30-yr-old groups (coefficient variations are 10.54%, others range 20.05 to 24.88%). There is no obvious tendency that leaf length increases or decreases with increasing tree age. Leaves in 30-yr-old are shorter than other age groups, especially significantly different from 13-yr-old tree age leaves (4.024 ± 0.424 cm and 4.887 ± 1.169 cm, *p* < 0.05). Leaves in 5-yr-old trees also significantly shorter than 13-yr-old trees (3.977 ± 0.904 cm, *p* < 0.05, more details are shown in Table 1).

**Fig. 3:**
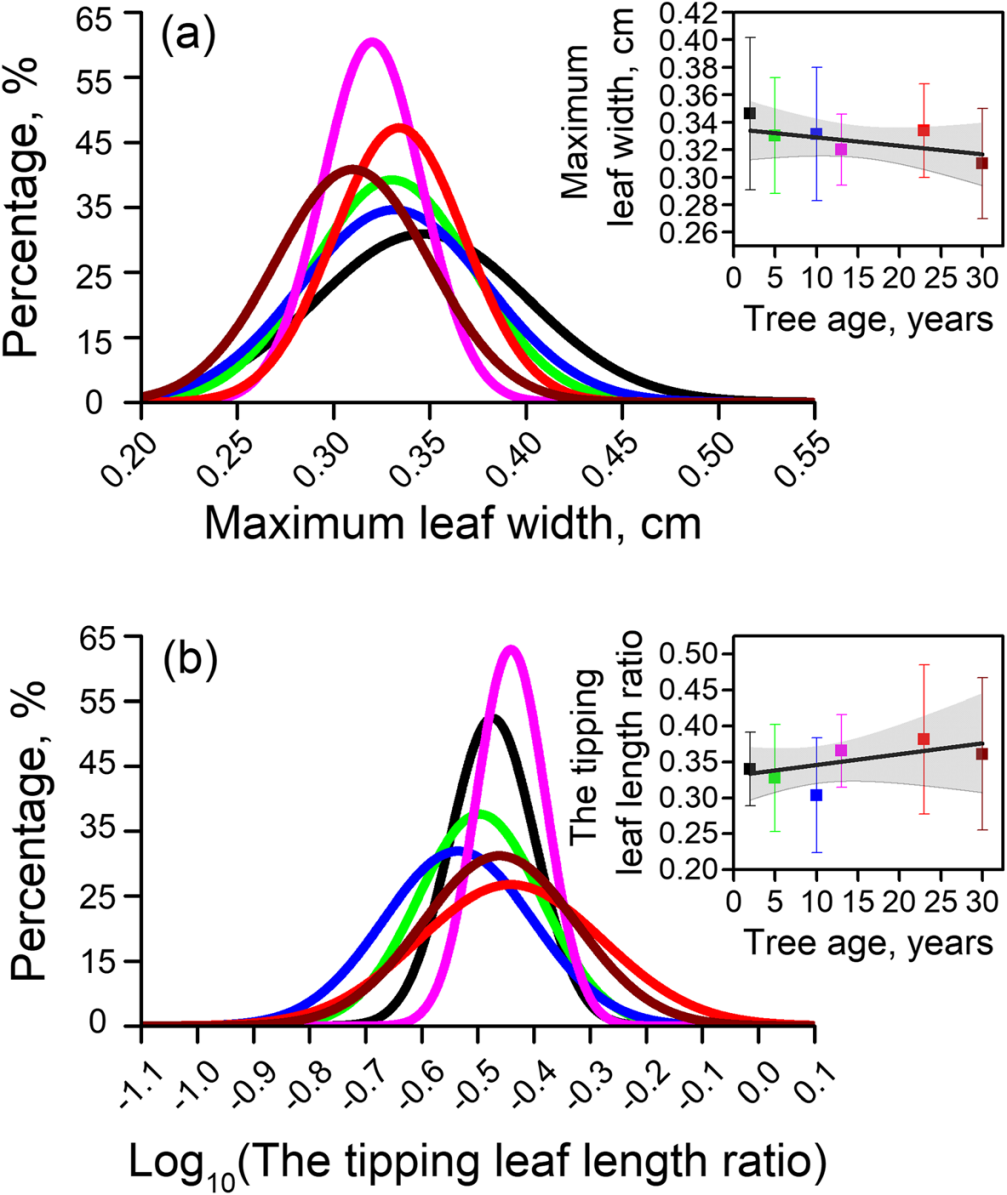
Normal distribution curves of (**a**) maximum leaf width range and (**b**) tipping leaf length ratio among different tree age groups. Differences between leaf width and leaf length ratio in each leaf along tree age groups were tested using a One-Way ANOVA test (Fisher LSD test) of significance. The statistical frequency distributions of leaf width and tipping length ratio across tree age groups are presented in supporting information Fig. S2 and Fig. S3 separately

Maximum leaf width averages in 0.329 ± 0.043 cm, and shows a decreasing trend with trees growth stages (from 0.346 ± 0.055 cm in 2-yr-old trees to 0.242 ± 0.084 cm in 30-yr-old trees, Fig. 3a, Table 1). Maximum leaf width in 2-yr-old trees are significantly larger than in 30-yr-old trees, while there are no significant differences among 5-, 10-, 13- and 23-yr-old trees (0.330 ± 0.042 cm, 0.331 ± 0.048 cm, 0.320 ± 0.026 cm, 0.334 ± 0.034 cm, respectively).

The measured data is fitted to determine the ratio between widest parts of leaf length from basal with total leaf length. The ratio floats in a certain range, varying from 0.303 for 10-yr-old trees to 0.381 for 23-yr-old trees, and averages 0.347 across tree age groups (Fig. 3b). The ratio decreases from 2-yr-old to 10-yr-old, then increases to 23-yr-old, finally decreases after 23-yr-old as a whole. Moreover, 10-yr-old trees show significant lower ratios than 13, 23, and 30-yr-old trees (0.365 ± 0.05 and 0.361 ± 0.106 for 13 and 30-yr-old trees length ratio, *p* < 0.05), and 23-yr-old trees are significant higher than 5-yr-old (0.328 ± 0.07) likewise (more details are shown in Table 1).

## Discussion

### The relation of leaf length and width

We pioneered a tapering model to intuitively describe the leaf length and width trajectory. Most studies were concentrated on measured leaf mean width and length, and their highly positive correlation (r > 0.5) [24]. Recently, many studies have proved that the relationship between leaf length and width is significant and implies evolutionary stability [11, 17]. Taken *C. lanceolata* as an example, we evaluated the model among sapling, juvenile, mature, and old trees. Before maturity, the period of leaf expansion was longer in juvenile trees than in the younger trees. That is consistent with leaf expansion tendency in angiosperms (*Euphorbiaceae*), which holds that leaf support tissues are widespread in early allocation for establishing hydraulic and mechanical infrastructure and preparing for further photosynthesis [39, 40]. Moreover, we supplement the follow-up leaf elongation in mature and old trees, in which leaves become shorter and wider compared with saplings and juveniles, as reported for Douglas-fir, an evergreen needle trees with dramatically morphological differences in saplings and old-growth [41].

### The specific parameters - leaf tipping length ratio

Further, we use the leaf tipping length ratio to evaluate the predictive power and applicability of the model about buffering strategy in growing stages. Most studies preferred to link environmental gradients with leaf patterns such as leaf width or the ratio between leaf length and width [24, 42]. Such relationships are often much conflicting against in different areas and floras [42, 43]; additionally, the parameters are not very typical for leaf hydraulic buffering strategy in growing stages. Because trees vascular evolution by natural selection has acted to minimize hydrodynamic constraints, showing sufficiently or moderately tapering from base to tip length to buffer hydraulic resistance [3, 44, 45]. The tipping ratio on the basal part (the widest represents the highest radial resistance) length to maximum hydraulic possibility length is more representative to explore the buffering strategy in leaves. The estimated mean ratio (0.347) of *C.lanceolata* is close to the theoretical value of 2/5 in ovate leaf, whose pattern is similar with elliptical in the basal part and parabolic in the distal part but much wider [26].

### The results of the parameter (leaf tipping length ratio) variations

The leaf length tipping ratio of *C. lanceolata* floats in a certain range during their developmental sequences. The theoretical value of ideal leaf pattern in each case for particular species provides an example to describe and distinguish plant categories [26]. Yet, the certain range of ratio indicates leaf hydraulic buffering strategy to support the best possible adaptive modification of the pre-existing hereditary type in different growth stages. For instance, our measured data and models generally state that leaves with decreasing tipping length ratio and increasing width tapering by trees maturing. When trees are mature (23-yr-old), they show a highest tipping length ratio (a larger basal part or a reduction of acuminate leaf tips), finally decline in old age. The results are similar to the former studies which reported from maize, tropical leaves, and conifers in Italy [9, 46–48]. The results also emerge as a signal that there possibly are functional constraints, biomechanical or physiological, operating in hydraulic buffering during growth process.

### The indication of the parameter variations -- biomechanics

From the view of leaf biomechanics, wider leaves require a greater midrib component and its increase is consistently associated with divergence in leaf width [24]. Indeed, the leaf tapering operates as a cantilevered beam and, further, provides economical mechanical support, due to the load is on the most basal part [15, 49]. The subtle fluctuation of the tipping length ratio proves that leaves can allow specialized biomechanical function to always ready to buffer different emergencies, such as wind, sun, and drought during their growth stages [50–52]. For example, as *C.lanceolata* and other long grass leaves exhibit, shifting more biomass to the distal part allows leaves bending so that the center of the leaves is faced towards sunlight in the prime (10-yr-old), or more ratio to the basal part bears more environmental supports when plant toward ageing (23-yr-old) [15, 46]. Besides, the ratio may increase with tree age or tree height, and the distal part is assumed to function as a way to quickly drain water from leaf surface and facilitate rapid drying [48]. Such function is reported as an adaptive strategy for tropical leaves [9].

### The indication of the parameter variations -- physiology

The change of growth rate and physiological process may be another constraint in hydraulic buffering. Leaf growing in full sun and water is often limited by the ability of biophysical architecture to maintain sufficiently hydrated [53, 54]. The tipping ratios are small in *C.lanceolata* saplings and juveniles, especially smallest in 10-yr-old trees, then increase to 23-yr-old in largest, finally decline in old age. Such trends are similar to the anatomical studies that reported single-vein leaves vascular cylinders area variation for Douglas-fir [41]. *C.lanceolata* mostly lives under conditions of abundant light and water, while they develop shaded in the understory in early stages, herein, act as activism with much consideration on efficiency. As we find that sapling (younger than 10-year-olds) individuals show a relative low tipping ratio and wider leaves, existing a relative high value tapering. The smaller leaf tipping ratio is supposed to indicate the higher leaf hydraulic conductance. Because the lower tipping ratio shows the high values of leaf width and vein tapering, and the ‘overtapering’ conduit structures lead to the total hydraulic resistance being lower [44, 47]. Additionally, the lower tipping ratio of *C.lanceolata* in elevated stages also entails higher gas exchange rate and higher relative growth rate for a given level of leaf carbon allocation [8, 15, 55, 56]. In such case, a general trend of increased leaf size in plants suggests that they prefer economic benefits of larger leaf area to capture more light rather than biomass allocation [57–59], and offer their root and vascular system architecture for tree height and chest developments, reducing the proportion of leaf mechanical support [60, 61]. Single-vein leaves can increase in size only by increasing the distal part due to the hydraulic constraint on maximum width, and tapering improves hydraulic capacity relative to cost [30, 31, 50, 62]. WBE (West, Brown, Enquist) model holds that sufficient tapering in vascular system can offset path-length-reduced resistance in sapling samples [3], and further studies found hydraulic resistance was predicted to increase slightly with path length, inducing moderate tapering from saplings to adults [44, 63, 64]. Moreover, plant architecture and hydraulic system have existed as a self-similar fractal. We add to prove that leaves show the same tapering trend in samplings and juveniles. Moreover, according to the hydraulic vulnerability segmentation hypothesis, the larger distal parts of xylem pathway allow to buffer more basal part from hydraulic failure such as cavitation events, and therefore supply water is less expendable [65, 66]. Therefore, for *C.lanceolata*, leaf hydraulic conductance may be high and rapid phases in the early, but slowdown growth in juveniles, and remain stable in established trees, moreover, an increase in old-growth. This can be proved from physiological studies, which believe plants greater access to water with deeper roots and attain powerful photosynthetic capacity with adequate photosynthetic area in the growing periods [14, 19], as well as it follows that plants have plenty of water supply to enable the stomata to remain open during the day [67, 68]. More construction in wider vein with narrower leaf width favors mature individuals that do not wastefully allocate carbon or nitrogen [69–71], after their golden age, they transit to conservatism with a wider leaf and moderate cost-benefit ratio. Many reports have been managed to verify a transition of mature to old-growth plants on physiology [72–74]. A relative long hydraulic pathway from vein through mesophyll tissue and moderate hydraulic efficient ratio render a relatively low hydraulic capacity, thereby limiting gas exchange to prevent the development of damaging water deficits [75].

## Conclusion

Single-vein leaves have the simplest symmetric distribution and structural design, being regarded as an appropriate but less-focused sample to explore buffering strategy based on leaf morphology. We pioneered a tapering model to describe half-leaf symmetric distribution, which provided the trajectory of leaf length and width. The ratio of leaf basal part length to total length (called tipping leaf length ratio) were evaluated according to the model to investigate the tradeoff of leaves between highest axial transport capacity and radial resistance, and the changes of such tradeoff during tree growth stages, serving as the signal of buffering strategy, indicating developmental plastically superiorities. Our measured data and models generally state that leaves with decreasing tipping length ratio and increasing width tapering by trees maturing. When trees are mature (23-yr-old), they show the highest tipping length ratio (a larger basal part or a reduction of acuminate leaf tips), finally decline in old age. The results indicate that there possibly are functional constraints, biomechanical or physiological, operating in hydraulic buffering during growth process, besides, this could be manifested in leaf morphology. Using the leaf tapering model and tipping leaf length ratio, we can further extend the buffering strategy to other more complex leaf-shaped species, thoroughly investigating the tradeoff in leaf hydrodynamical, biomechanical and geometrical design during tree growth stages.

## Materials and methods

### The model

Previous have used many models to describe the leaf shape, for instance, the shape of bamboo leaves follows the simplified Gielis equation [18]. While for *C. lanceolata*, the model is aimed at simulating leaf length and width trajectory by single leaves, in terms of leaves geometric shapes which are ellipses and parabolas [26]. Parabolic model characterizes the half-leaf hydraulic possibility in terms of single leaf tapering shape (Fig. 1).

1 $$ Y=C+{B}_1X+{B}_2{X}^2 $$

(Y: Leaf half width, X: leaf length, C is constant representing intercept)

When $\frac{dY}{dX}=0$, where X_0_ is the position of length, the maximum Y is approached.

2 $$ {B}_1+2{B}_2{X}_0=0 $$

3 $$ {X}_0=-\frac{B_1}{2{B}_2} $$

(X_0_ is the maximum width in the leaf length position)

4 $$ Tipping\ leaf\ length\ ratio=\frac{X_0}{X_{max}} $$

(X_max_ is the leaf total length, while tipping leaf length ratio means the widest part of the leaf is on the axis in the basal ratio of the leaf)

### Leaf materials

All plant materials were obtained from the wild with authorization and permission from one of national forest research station in China.

We choose Chinese fir (*C. lanceolata*) tree species as an example to test our modeling framework. The field studies did not involve endangered or protected species according to Chinese law and the Trade in Endangered Species of Wild Fauna and Flora (https://cites.org/). *C. lanceolata* is a coniferous tree species endemic to China, being one of the major fast-growing, high-yielding, and high-quality timber species in south China [35, 36].

The study area was Huitong National Forest Ecosystem Research Station located in Huitong, Hunan province, China (25°50′N, 109°45′E). Experimental research on plants in long-term experimental stations and/or forest farms are encouraged according to policies and regulations of the state forestry administration in China and the outline of the national plan for medium- and long-term scientific and technological development. Organs of *C. lanceolata* were previously sampled and analyzed for several functional traits measured in young to mature-aged trees grown in wild at China forest station [36]. In the present study, we perform our investigation along tree growth development and across soil resource availability gradient. We obtained authorization and permission from the Huitong Forest Ecosystem Research Station to collect plant materials during the entire period of this study. Leave samplings and laboratory measurements were conducted in May 2018.

The site is dominated by a humid mid-subtropical monsoon climate, with warm and humid all year round. The annual mean temperature is 16.4 °C, and mean annual precipitation is 1270–1650 mm occurring mostly between April and August (the data was provided by the China Meteorological Data Network (http://data.cma.gov.cn/site/index.html) and based on 1987–2015 normal). Average relative humidity exceeds 90% while average annual sunshine hours over 1350 h [29]. The soil is haplic alisols/haplic acrisols in World Taxonomy (clay loam red earth in Chinese Soil Taxonomy) developed from shale and slate parent rocks. The second generation of *C.lanceolata* forest was replanted in 1987, with an initial stand density of 3318 trees hm^− 2^. The forest was nursed twice in spring and autumn in the first three years (1987–1990), and there was no tending and thinning management after 1990, which let the forest grow naturally [36]. The total forest density is about 2310 trees hm^− 2^ at present. Six age groups of *C.lanceolata* trees existing within the research station were selected which are saplings (2, 5, and 10-yr-old, occur naturally with no cultivation treatments), juveniles (13-yr-old), mature (23-yr-old), and old-growth individuals (30-yr-old). Needles were selected 6–8 *C.lanceolata* trees which were well-growing and disease-free comprising different depths of canopy in each stands. Sampling leaves were placed in plastic bags with wet tissue inside and delivered to the laboratory. We selected leaves that apparently differed in size and shape, and collected 308 leaves per age group stands and 1848 leaves in total. All leaves were used immediately for the determination of further measurements.

### Leaf measurements

All samples were scanned using an Epson Expression 11000XL Photo Scanner (Seiko Epson Inc., Tokyo, Japan), with the 600 dpi needed to clearly display leaf shape features was adopted (Fig. S1a). In order to acquire complete representations of leaf shape, sample images were with the help of a commercial graphic editor (AutoCAD, Autodesk Inc. San Rafael, CA, USA) to further clear backgrounds by smoothing and binary coding (Fig. S1b). A great effort was devoted to the image content digitization in this work. Data were obtained by applying a georeferencing framework with ARCGIS v9.3. (ESRI, Redlands, CA, USA) to the vector representation of leaf length and width based on artificial neural network (Fig. S1c) [69]. Leaf length was the distance between leaf base to blade tip along leaf midrib, and leaf length was divided into a number of sections per 0.1 or 0.2 cm, while leaf widths were the distances perpendicular to midrib at every leaf length intervals (Fig. 1).

### Statistical analyses

In order to simulate leaf length and half-leaf width distribution of *C. lanceolata*, we fitted leaf tapering function with parabolic models. The best 108 leaves were used to construct the leaf tapering models to calculate the average model in each age group trees and the remaining leaves were used to test the robust of the model and all leaves (308 leaves per age groups and 1848 leaves in total) are used in analysis. Means, standard deviation, 95% confidential interval, median, minimum and maximum value were used to describe leaf width and tipping length ratio. We have checked normality of all data (Shapiro-Wilk normality test), and the tipping leaf length ratio was log10-transformed to test differences between each age groups. The differences between leaf width and tipping length ratio in each leaf among tree age groups were tested using a One-Way ANOVA test (Fisher LSD test) of significance. The model fitting, statistical description and ANOVA test were performed in software IBM SPSS Statistics v 21.0 (IBM Corp., Armonk, NY, USA). The charts were plotted in software OriginPro v 8.0 (OriginLab Co., Northampton, MA, USA) and R software version 3.6.1 (R Core Team 2019) [76].

## Supplementary Information

**Additional file 1: Figure S1.** Data extraction processes of selected *C. lanceolata* leaves cross tree age stages ranging 2-yr-old to 30-yr-old, more details see Materials and Methods. (a) Original scanned image. (b) Smoothed and binary image. (c) Evaluation of leaf length and width by vectoring leaf margin using ArcGIS v 9.3. The diameters are shown in images. **Figure S2.** Leaf width statistical frequency distribution across tree age gradients of *C. lanceolata.* **Figure S3.** The tipping length ratio statistical frequency distribution across tree age groups of *C. lanceolata*. The plus and value showing in images represent mean value between groups.

**Additional file 2: Table S1.** Regression coefficients, R^2^, horizontal and longitudinal distance of apex, and the ratio between the horizontal distances of apex with total length of main relationships used to estimate the length and half-leaf width trajectories in the selected leaves. The best fitting on leaf length(X) and half-leaf width(Y) is obtained by quadratic-function models: Y=C + B_1_X + B_2_X^2^, and all regressions have *p* < 0.0001.

**Additional file 3.**

## Data Availability

Data are made available as supplementary material.
