# Supplementary material for: Half-leaf width symmetric distribution reveals buffering strategy of Cunninghamia lanceolata
Source: BMC Plant Biol. 2021 May 17;21:222. doi: 10.1186/s12870-021-03000-x (PMC8127188; doi:10.1186/s12870-021-03000-x)
Supplement: Supplementary file 1 — Additional file 1: Figure S1. Data extraction processes of selected C. lanceolata leaves cross tree age stages ranging 2-yr-old to 30-yr-old, more details see Materials and Methods. (a) Original scanned image. (b) Smoothed and binary image. (c) Evaluation of leaf length and width by vectoring leaf margin using ArcGIS v 9.3. The diameters are shown in images. Figure S2. Leaf width statistical frequency distribution across tree age gradients of C. lanceolata. Figure S3. The tipping length ratio statistical frequency distribution across tree age groups of C. lanceolata. The plus and value showing in images represent mean value between groups. [file 12870_2021_3000_MOESM1_ESM.doc]

## *BMC Plant Biology* Supporting Information

# Article title: Half-Leaf width symmetric distribution reveal buffering strategy of fast-growing species

# Authors:

# Article acceptance date:

# Supporting Information List

The following Supporting Information is available for this article:

## Figure S1


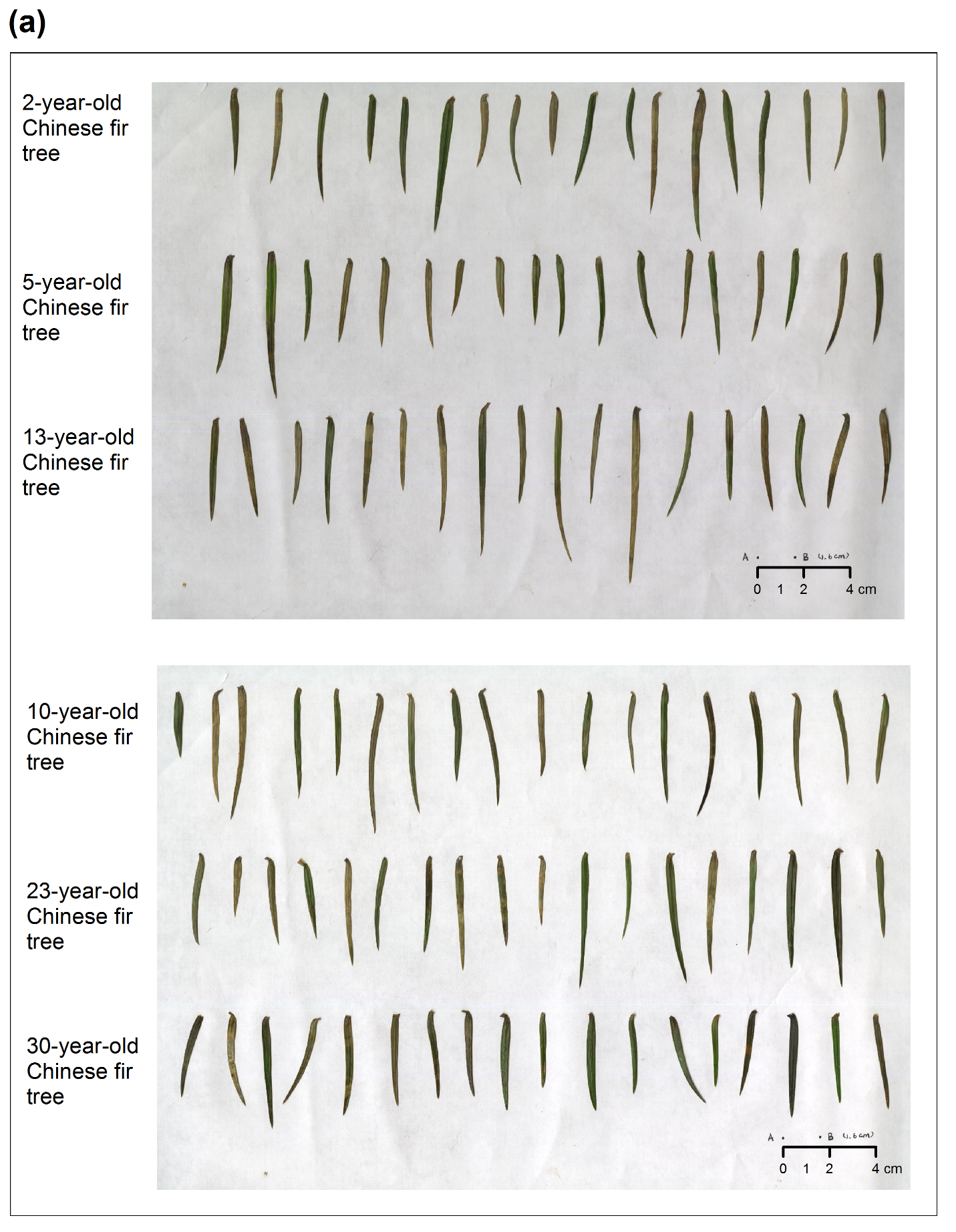


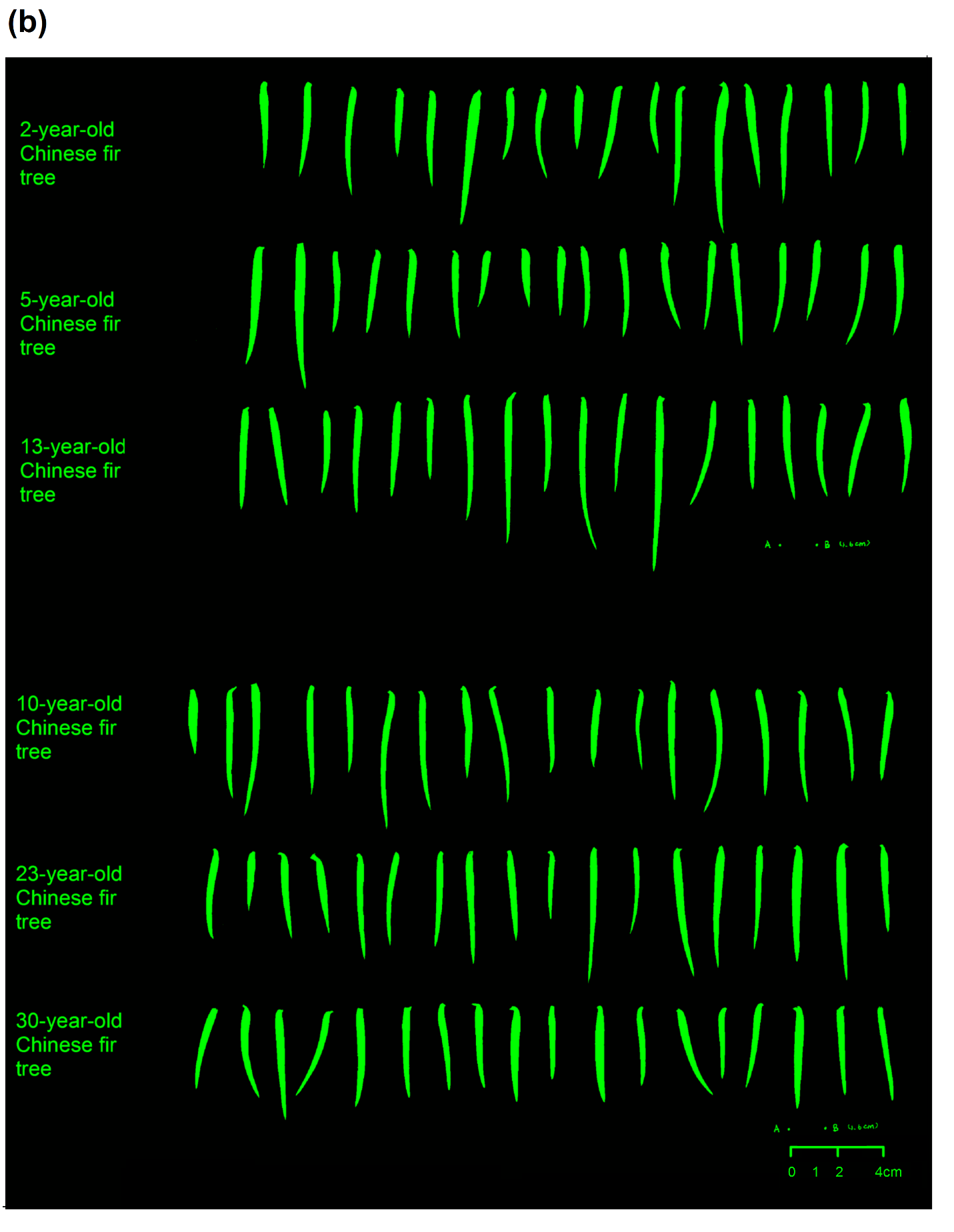


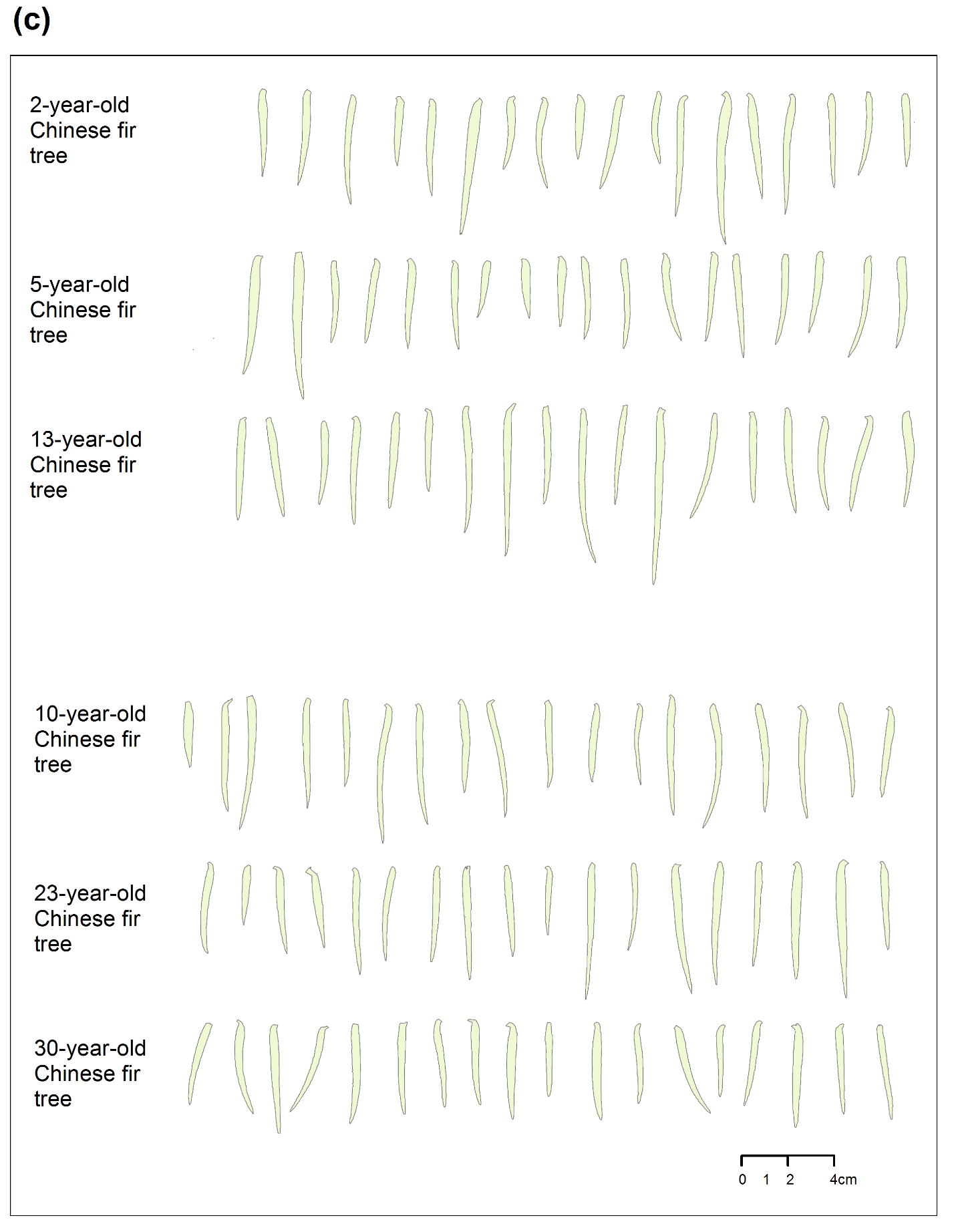


Figure S1. Data extraction processes of selected *C. lanceolata* leaves cross tree age stages ranging 2-year-old to 30-year-old, more details see Materials and Methods. (a) Original scanned image. (b) Smoothed and binary image. (c) Evaluation of leaf length and width by vectoring leaf margin using ArcGIS9.3. The diameters are shown in images.

## Figure S2





Figure S2. Leaf width statistical frequency distribution across tree age gradients of *C. lanceolata*.

## Figure S3





Figure S3. The tipping length ratio statistical frequency distribution across tree age groups of *C. lanceolata*. The plus and value showing in images represent mean value between groups.
